# Prevalence of Promotional Bias and Quality Concerns of LED Light Therapy Masks: A Cross‐Sectional Analysis on TikTok

**DOI:** 10.1111/jocd.70416

**Published:** 2025-08-25

**Authors:** Manas Ranpariya, Purvi Shakelly, Madeline Tchack, Bassem Rafiq, Babar K. Rao

**Affiliations:** ^1^ Rutgers Robert Wood Johnson Medical School New Brunswick New Jersey USA; ^2^ Rutgers New Jersey Medical School Newark New Jersey USA; ^3^ Center for Dermatology Rutgers Robert Wood Johnson Medical School Somerset New Jersey USA; ^4^ Rao Dermatology Atlantic Highlands New Jersey USA

**Keywords:** acne cosmetics, cosmetic dermatology, dermatology, light emitting diode (LED), skin care

To the Editor,

TikTok has rapidly emerged as a leading platform for dermatology‐related content, delivering educational insights, general information, and serving as a powerful marketing tool. However, the reliability of health information on the app remains a question of study [1]. Light‐emitting diode (LED) light therapy masks, marketed for treating cosmetic skin concerns like acne, hyperpigmentation, and signs of aging, are prominently featured on TikTok videos. These devices are promoted as a convenient alternative to clinical LED light panels commonly used in dermatology practices, aiming to stimulate collagen production and other skin‐rejuvenating processes through targeted light wavelengths. However, the irradiance and dosage levels in at‐home LED masks are typically much lower than those of clinical‐grade panels, potentially diminishing efficacy [2, 3]. Additionally, although risks are not yet fully understood, there have been reports of eye damage, skin irritation, and cutaneous lupus erythematosus‐like reactions, especially with improper use [4].

Given their popularity, evaluating the credibility of information on TikTok about LED therapy masks is essential to aid informed patient–physician discussions. This study aims to assess the viewership, quality, and creators of TikTok videos on LED light therapy masks to identify trends of misleading or unsubstantiated information.

On October 15, 2024, TikTok was queried using the search terms “LED Light Therapy Masks” to collect the top 100 posts as determined by TikTok's sorting algorithm. Non‐English videos and those unrelated to LED light therapy masks were excluded. Post data was categorized by author type: physicians, non‐physician providers (NPP), influencers (non‐healthcare associated individual accounts with > 5000 followers), and others. Two independent reviewers rated the quality of each video with the DISCERN instrument which is a series of sixteen 5‐point Likert scale questions with scores ranging from 15 to 80 (higher scores indicating greater quality) [5]. Average scores between the two reviewers were calculated for analysis.

Our analysis revealed that influencer accounts dominated the sample, with 63 out of 100 total included posts. Promotional content was the most prevalent content category among NPPs (71.4%), influencers (80.1%), and others (80%). Educational content was proportionally more prevalent among physician accounts (62.5%). Interrater reliability was high for DISCERN scores (Cohen's weighted kappa > 0.75). Physician posts had the highest mean DISCERN scores (25.56), while influencer videos scored the lowest (19.76), indicating an inclination toward higher‐quality content from physicians (Table 1).

**TABLE 1:** TikTok post data categorized by creator type.

	Physician	NPP	Influencer	Other
No. of accounts (*n*)	8	14	63	15
Mean likes	20 769	44 014	13 156	2159
Mean comments	320	344	144	49
Mean shares	1258	2097	584	197
Content category, *n* (%)				
Educational	5 (62.5%)	4 (28.6%)	4 (6.4%)	2 (13.3%)
Promotional	3 (37.5%)	10 (71.4%)	51 (80.1%)	12 (80%)
Personal	0	0	8 (12.7%)	1 (6.7%)
Mean discern score	25.56	23.61	19.76	20.43

This study identifies a concerning imbalance in TikTok content related to LED light therapy masks, where promotional posts—particularly from non‐physicians and laypersons—frequently overshadow evidence‐based educational material. TikTok's affiliate marketing infrastructure, including its integrated store feature, incentivizes creators to prioritize advertisement‐driven content, often at the expense of accuracy and safety. Posts rarely mention contraindications, precautions, or clinical evidence, potentially fostering unrealistic expectations and improper use. The platform's algorithm, which favors engaging and viral content, likely amplifies these promotional trends, further diminishing the visibility of reliable health information. As LED masks gain traction for their perceived convenience and affordability compared to professional treatments, the scarcity of accessible, evidence‐based content poses risks to informed consumer decision‐making. Study limitations include a small sample size (100 posts), content collected on a single day, and potential exclusion of relevant material due to search term variability. Future research could benefit from larger, longitudinal datasets and expanded search strategies to better characterize content trends.

## Author Contributions

M.R. and P.S. performed the research and wrote the manuscript. M.T., B.R., and M.R. contributed to the study design and assisted in refining the research methodology. B.K.R. served as the principal investigator and provided overall supervision. All authors have read and approved the final manuscript.

## Consent

The authors have nothing to report.

## Conflicts of Interest

The authors declare no conflicts of interest.

## Data Availability

The data that support the findings of this study are available from the corresponding author upon reasonable request.
